# METABOLOMIC AND IMMUNOLOGIC INSIGHTS INTO RACIAL DIFFERENCES IN REPRODUCTIVE AGING

**DOI:** 10.1093/geroni/igae098.2628

**Published:** 2024-12-31

**Authors:** Joanna-Lynn Borgogna, Johanna Holm, Jacques Ravel, Rebecca Brotman, Jovanka Voyich, Carl Yeoman, Michelle Shardell

**Affiliations:** Montana State University, Bozeman, Montana, United States; University of Maryland School of Medicine, Baltimore, Maryland, United States; University of Maryland School of Medicine, Baltimore, Maryland, United States; University of Maryland School of Medicine, Baltimore, Maryland, United States; Montana State University, Bozeman, Montana, United States; Montana State University, Bozeman, Montana, United States; University of Maryland School of Medicine, Baltimore, Maryland, United States

## Abstract

Menopause is linked to declining levels of estrogen, loss of protective vaginal lactobacilli, and age-related comorbidities. Vaginal metabolites reflect metabolic activity of the microbiota and human host, along with exogenous influences, and can modify susceptibility to multiple pathologies. Racial/ethnic minorities experience earlier and more intense symptoms of menopause but are underrepresented in reproductive aging studies. To address these gaps, we applied 16S rRNA gene sequencing, untargeted metabolomics, and cytokine profiling to characterize the vaginal microenvironment across reproductive stages, with an emphasis on comparisons between post-menopausal racial/ethnic groups. 476 participants (aged 35-60 years) contributed semiannual person-visits over two years (N=1,153 samples). Bayesian mixed-effects regression of log2-transformed metabolites (n=770) assessed metabolomic differences in samples from pre- (n=287), peri (n=335), and post-menopausal (n=531) participants, 25% of whom were racial/ethnic minorities. Samples collected from post-menopausal participants had distinct metabolomic profiles compared to pre- or peri-menopausal samples (548 metabolites were lower in post-menopausal samples; p< 0.05). After adjustment for vaginal microbiota, post-menopausal samples had lower concentrations of most micronutrients (e.g., vitamins B, C, and D; p< 0.05). Metabolites reflecting epithelial damage and oxidative stress, along with host-produced lysophospholipids (e.g., lactosyl-N-palmitoyl-sphingosine), were highest in samples from post-menopausal participants (p< 0.05), with post-menopausal Black participants having the highest levels (relative to post-menopausal white participants, and non-postmenopausal Black or white participants) (p< 0.05). Lysophospholipids are immunogenic, and many were positively correlated with TNF-α, but negatively correlated with IL-19. Further investigations linking menopause and race/ethnicity with inflammatory or oxidative stress-related metabolites are needed to reduce disparities in healthy reproductive aging.

